# Data-driven visualization of multichannel EEG coherence networks based on community structure analysis

**DOI:** 10.1007/s41109-018-0096-x

**Published:** 2018-09-26

**Authors:** Chengtao Ji, Natasha M. Maurits, Jos B. T. M. Roerdink

**Affiliations:** 1 Bernoulli Institute for Mathematics and Computer Science and Artificial Intelligence, University of Groningen, Nijenborgh 9, Groningen, 9747AG The Netherlands; 2 Department of Neurology, University Medical Center Groningen, University of Groningen, Hanzeplein 1, Groningen, 9713GZ The Netherlands; 3 Neuroimaging Center, University Medical Center Groningen, University of Groningen, Hanzeplein 1, Groningen, 9713GZ The Netherlands

**Keywords:** Multichannel data, Community structure, Visualization, Brain connectivity

## Abstract

An electroencephalography (EEG) coherence network is a representation of functional brain connectivity, and is constructed by calculating the coherence between pairs of electrode signals as a function of frequency. Typical visualizations of coherence networks use a matrix representation with rows and columns representing electrodes and cells representing coherences between electrode signals, or a 2D node-link diagram with vertices representing electrodes and edges representing coherences. However, such representations do not allow an easy embedding of spatial information or they suffer from visual clutter, especially for multichannel EEG coherence networks. In this paper, a new method for data-driven visualization of multichannel EEG coherence networks is proposed to avoid the drawbacks of conventional methods. This method partitions electrodes into dense groups of spatially connected regions. It not only preserves spatial relationships between regions, but also allows an analysis of the functional connectivity within and between brain regions, which could be used to explore the relationship between functional connectivity and underlying brain structures. As an example application, the method is applied to the analysis of multichannel EEG coherence networks obtained from older and younger adults who perform a cognitive task. The proposed method can serve as a preprocessing step before a more detailed analysis of EEG coherence networks.

## Introduction

EEG records the electrical activity of the brain by attaching electrodes to the scalp of a subject at multiple locations. Synchronous electrical activity in brain regions is generally assumed to imply functional integration. Such synchronization occurs over a large range of scales. For example, local synchronization occurs during visual processing, synchronization between neighboring temporal and parietal cortical regions is observed during multimodal semantic processing, and long-range fronto-parietal interactions occur in working memory retention and mental imagery (von Stein and Sarnthein 2000). A large number of methods have been proposed to measure the synchrony between pairs of brain regions, and these measures are often closely correlated (Nunez et al. 1997). EEG coherence is one of these measures, which is calculated between pairs of electrode signals as a function of frequency (Halliday et al. 1995; Maurits et al. 2006).

Visualization provides a visual representation of the data to help people carry out analysis tasks effectively. It happens at an early stage in the process, usually before a statistical or computational analysis, and it allows people to explore their data before knowing exactly what kind of questions to ask (Munzner 2014). After the questions have been well-defined, existing methods such as machine learning can be used to answer them. For the case of brain connectivity, visualization can lead to the discovery of unanticipated patterns (Alper et al. 2013), thus providing insight into brain functioning. In this way it can help neuroscientists to understand how the brain works, especially for the case where no a priori assumptions or hypotheses about brain activity in specific regions are made (Ma et al. 2015; Li et al. 2012; Xia et al. 2013; Christodoulou et al. 2011; Gerhard et al. 2011; Fujiwara et al. 2017).

**Fig. 1:**
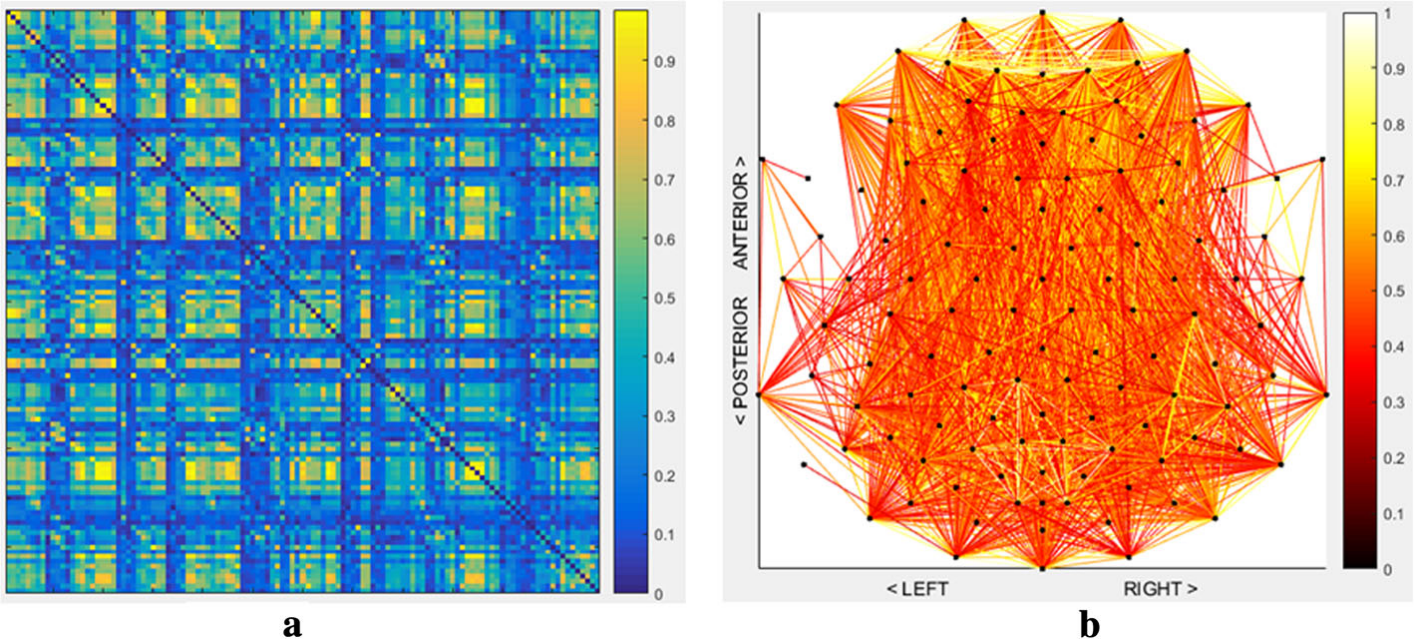
Convention types of EEG coherence network visualization techniques. **a** Matrix Representation. **b** Node-link diagram

An EEG coherence network represents functional brain connectivity, more precisely, the coherences between pairs of signals recorded by the electrodes. However, visualization of high-density or multichannel EEG (at least 64 electrodes) coherence networks is not always managed well (ten Caat et al. 2008). Typical visualizations of coherence networks are a matrix representation with rows and columns representing electrodes with cells representing coherences between electrode signals or a node-link diagram with vertices representing electrodes and edges representing coherences (see Fig. 1). Edges are considered significant when their coherence passes a significance threshold (Halliday et al. 1995), see also the description of the experimental setup in the “Results” section. However, such representations can suffer from some drawbacks. The matrix representation is very effective for visualizing large and/or dense networks (Alper et al. 2013; Yi et al. 2010), but the relative spatial location of electrodes is hard to embed in this matrix. Therefore, brain connectivity network visualization is complex. The common focus on specific connections is often insufficient to explain all aspects of information contained in the network, because the spatial context of the connections is also crucial in the analysis of brain connectivity. In contrast, the node-link diagram is a straightforward method of visualizing networks that preserves spatial information about the electrodes well. This visualization, however, suffers from the potentially large number of overlapping edges when visualizing dense networks, which makes it hard to distinguish connections (Kamiński et al. 1997).

To study connectivity patterns in the coherence graph, researchers often employ a hypothesis-driven or semi-data-driven definition of certain regions of interest (ROIs), in which all electrodes are assumed to record similar signals because of volume conduction effects (Gladwin et al. 2006; Lachaux et al. 1999). However, these methods generally depend on certain assumptions or hypotheses, where an a priori selection of relevant electrodes is made. As a consequence, not all the available data is used and the underlying brain network is not fully explored.

As an alternative to the hypothesis-driven and semi-data-driven approaches, ten Caat et al. (2007a,, 2008) proposed a method for detection of data-driven ROIs, referred to as *functional units* (FUs). An FU is represented in the coherence graph by a spatially connected maximal clique (a clique is a vertex set in which every two-element subset is connected by an edge). Because larger ROIs are assumed to correspond to stronger source signals, larger FUs are considered to be more interesting. Therefore, the maximal clique based (MCB) method of ten Caat et al. (2007a) focused on *maximal* cliques, for which vertex sets are as large as possible. Since the MCB method is very time-consuming (time complexity *O*(3^*n*/3^), with *n* the number of vertices), as an alternative a watershed based (WB) method was proposed that detects spatially connected cliques in a greedy way (ten Caat et al. 2007b). To reduce the potential over-segmentation of this approach, an improved watershed based method (IWB) (time complexity *O*(*n*^2^ log*n*)) was proposed that merges FUs if they are spatially connected and if their union is a clique (ten Caat et al. 2008). The WB and IWB methods are up to a factor of 100,000 faster than the MCB method for a typical multichannel setting with 128 EEG channels, thus making interactive visualization of multichannel EEG coherence possible. The FUs detected by the (I)WB method are similar but not identical to the FUs detected by the MCB method. To distinguish them we therefore denote the corresponding FUs by FU^MCB^ and FU^IWB^, respectively.

To visualize the FUs, ten Caat et al. (2007a) introduced the concept of *FU map*, which represents the FUs as a set of Voronoi cells (one for each electrode) with identical gray value, with different gray values for adjacent FUs (see Fig. 7 in the “Method” section for an example). A color-coded circle is placed at each FU center that represents the average coherence of this FU, and a color-coded line is drawn between pairs of FU centers if the average coherence between these two FUs exceeds the coherence threshold. This visualization shows both synchronization within and between FUs.

A drawback of the MCB and IWB methods is that the analysis of local synchronization is difficult, since these methods detect maximal cliques, that is, groups of spatially-connected electrodes that are as large as possible. Specifically, the MCB method views coherences above or equal to the pre-defined threshold equally while the IWB method clusters electrodes based on their neighbours which have the largest coherence value with them, without considering how strong the electrodes are connected with the other members of the group. Therefore, in this paper we propose an alternative method, based on detecting densely interconnected groups of brain regions known as network *communities*, which can be observed either in anatomical or in functional networks (Honey et al. 2007; Rubinov and Sporns 2010). The composition of these individual groups, known as *community structure*, is usually obtained by optimizing the *modularity* measure, as proposed by Newman (2004). We use the community structure analysis of a network as a data-driven approach to facilitate the visual analysis and interpretation of multichannel EEG coherence networks. The proposed method, referred to as the community clique-based (CCB) method, partitions the set of electrodes into several data-driven ROIs (communities) based on their connections and positions. As a result, electrodes within the same community are spatially connected and are more densely connected than electrodes in different communities. For brevity, the ROIs obtained by community detection will still be called “functional units”, but denoted by the symbol FU^CCB^ to distinguish them from the functional units obtained by the MCB and IWB methods. The functional units detected by the CCB method are visualized in an FU^CCB^ (functional unit) map, in exactly the same way as for the FU^MCB^ and FU^IWB^ maps.

A preliminary version of this paper appeared in Ji et al. (2018). In that paper, we briefly described the new method, and compared it with the other two methods. In this paper, we provide more algorithmic details about the three methods, compare the three methods when applied to synthetic and real EEG coherence networks, and apply the new method in one example application. This application shows the potential of our method for visualizing multichannel EEG coherence networks.

Overall, the new community-based method for detecting functional units is not only expected to reduce the drawbacks of the conventional hypothesis-based approaches, but also to allow a more detailed analysis of the relationship between functional connectivity and underlying brain structure than the data-driven MCB and IWB methods.

## Related work

The principal concept in our approach is to visualize brain connectivity, and to extract meaningful information from this representation for further analysis. The challenge in visualization often lies in the analysis of a huge amount of data, in our case the large number of EEG channels.

A straightforward method would be to visualize functional brain connectivity data as 3D node-link diagrams: ROIs are shown as nodes and the relationships between these nodes are encoded in the edges. But this approach suffers from visual clutter, and side effects of 3D rendering such as occlusion are hard to remedy (Alper et al. 2013; Dosenbach et al. 2010).

**Fig. 2:**
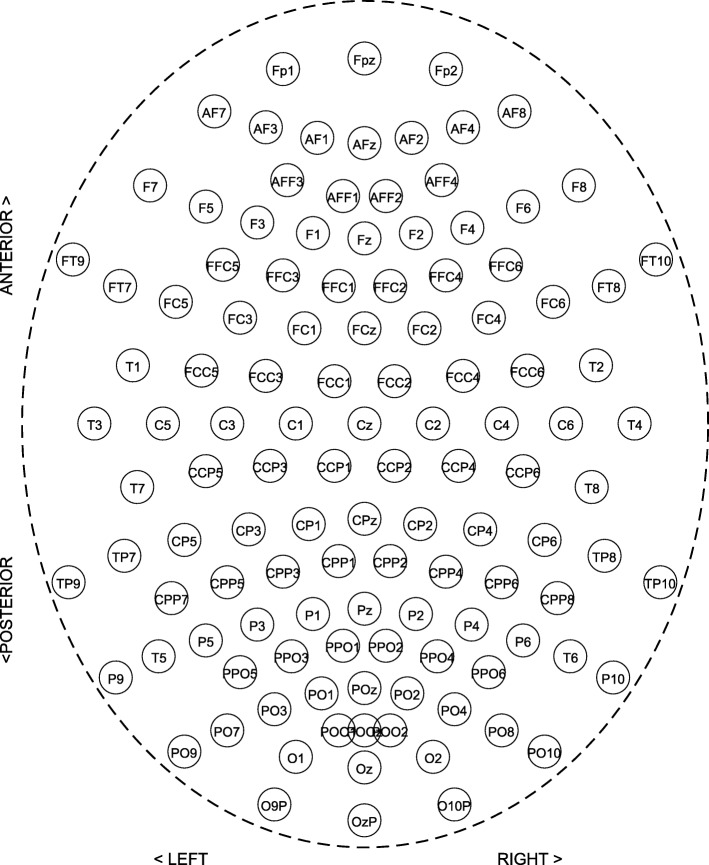
Electrode positions and labels in the 10-20 system Oostenveld and Praamstra (2001). Black circles indicate positions of the original 10-20 system, gray circles indicate additional positions introduced in the 10-10 extension. Each site has a letter to identify the general brain region and a number to identify the hemisphere location. The letters, Fp, F, T, C, P, and O stand for fronto-polar, frontal, temporal, central, parietal, and occipital, respectively. For further explanation of the 10-20 system, see Oostenveld and Praamstra (2001)

An alternative approach is to depict the connectivity data by a 2D representation, which could reduce the work of 3D rendering. A wide variety of methods has been developed to map data on 2D space to visualize neuronal interactions or relations between brain regions. To preserve the spatial information of the data to some extent, a node-link diagram based on a biologically meaningful layout has been used (Achard 2006). In this biological layout, planar projections are used for the 3D electrode locations on the surface of a head (see for example Fig. 2). Vertices are usually mapped according to a top view of the head, sometimes to two separate side views of the left and right hemispheres. However, such a visualization with edges representing connections may suffer from a large number of overlapping edges, resulting in a cluttered representation, especially for a large amount of data.

Some methods were proposed to remedy the visual clutter by eliminating overlaps and reducing the number of long-distance edges employing graph drawing. For example, for 2D node-link diagrams the layout can be calculated by multidimensional scaling or force-directed algorithms (Fruchterman and Reingold 1991). However, such methods usually change the layout of the vertices to reduce visual clutter. Yet, the spatial context of the data is still vital for facilitating the interpretation of the data by neuroscientists, who are trained in reasoning with respect to spatial brain regions. Hence, node-link diagrams are often accompanied by a separate picture showing the position of nodes, with nodes on the two representations being matched by color encoding or labeling (Nelson et al. 2010). In this approach, information about spatial context is not presented in a single image. Matrix representations are also popular to represent functional connectivity networks. This approach outperforms the node-link diagram in visualizing large networks. By arranging ROIs along the rows and columns of a matrix, their spatial relations are, however, lost (Kamiński et al. 1997).

Some studies explored the capabilities of node-link diagrams and matrix representations, and these suggested that the matrix representations outperform node-link diagrams for most tasks when the number of nodes becomes very large (Alper et al. 2013; Ghoniem et al. 2005).

Based on the basic node-link and matrix representations, several other useful tools have been developed for visualizing brain networks. To take advantage of both node-link diagrams and matrix representations, some visualization methods provide a dual representation to explore networks (Ma et al. 2015; Henry and Fekete 2006). Christodoulou et al. present BrainNetVis to support brain network modelling and visualization by providing both quantitative and qualitative network measures of brain connectivity (Christodoulou et al. 2011). Gerhard et al. introduce the Connectome Viewer Toolkit for connectome mapping, analysis, and visualization (Gerhard et al. 2011). Xia et al. present BrainNet Viewer to display brain surfaces, nodes and edges as well as properties of the network (Xia et al. 2013). Fujiwara et al. introduce a visual analytics system for comparison of brain connectivity across individuals, groups, and time points (Fujiwara et al. 2017). However, none of these methods can completely overcome the intrinsic drawbacks of node-link diagrams and matrix representations. To present brain connectivity in a single image and overcome the drawbacks of conventional methods, we reduce the visual clutter by putting nodes with specific properties, based on their spatial information and topological network properties, into the same group. This presentation then serves as a first step before a more detailed analysis of EEG coherence networks.

## Method

Although there are many unsupervised graph clustering methods, they either aim to find a predetermined number of clusters or do not consider the spatial information of the data (Ahmadlou and Adeli 2011; Kirschner et al. 2012; Schaeffer 2007). ten Caat et al. proposed a method considering the functional connections and spatial information of nodes together (ten Caat et al. 2008). This method assumes that a spatially connected set of electrodes records similar signals as a result of volume conduction (ten Caat et al. 2008; 2007a). However, one potential drawback of this method is that it is not easy to analyze local synchronization since the method tries to find groups of electrodes that are as large as possible.

In this section, we first provide some background on EEG coherence and the data representation of the EEG coherence network. Then we describe the community clique-based method of detecting dense spatially-connected groups of electrodes. Then, we briefly describe the MCB and IWB methods for later comparison in the “Results” section.

### EEG coherence

During an EEG experiment, brain activity is recorded by electrodes attached to the scalp of a subject at different locations. The electrodes are placed based on the 10/20 system and the position of an electrode is indicated by its label which is a combination of letters indicating brain regions (e.g., F for frontal), and digits indicating lateralization (odd numbers for left, even for right) and distance from the midline (higher numbers are farther away) (see Fig. 2) (Luck 2005; Oostenveld and Praamstra 2001). A conductive gel is applied between electrodes and skin for reducing impedance. EEG provides high-resolution temporal information about brain activity, and the electrical potential is typically measured at sampling rates up to 2000Hz. The measured signal that is recorded from each site is amplified, resulting in one recording channel for every electrode. If there are many electrodes, e.g., 64 or 128, the term “multichannel” or “high-density” EEG is used.

Activity from one source can result in a strong signal recorded by multiple electrodes, and nearby electrodes usually record similar signals due to volume conduction and reference electrode effects (Holsheimer and Feenstra 1977; Nunez et al. 1997). Often, there are several sources of activity at different locations, and these sources can be synchronized. Consequently, signals recorded by electrodes that are far apart can also be similar. The degree of interaction between two signals can be measured by coherence which is a measure for the similarity of signals as a function of frequency.

The coherence *c*_*λ*_ as a function of frequency *λ* for two continuous time signals *x* and *y* is defined as the absolute square of the cross-spectrum *f*_*xy*_ normalized by the autospectra *f*_*xx*_ and *f*_*yy*_ (Halliday et al. 1995), having values in the interval [0, 1]: $c_{\lambda }(x,y) = \frac { |f_{xy}(\lambda)|^{2} } {f_{xx}(\lambda)f_{yy}(\lambda)}$.

### Data representation and EEG coherence network

A network is simply a collection of connected objects. We refer to the objects as nodes or vertices and the connections between the nodes as edges. In mathematics, networks are often referred to as *graphs*, and the area of mathematics concerning the study of graphs is called *graph theory*. In this paper, we use the terms *network* and *graph* interchangeably.

**Fig. 3:**
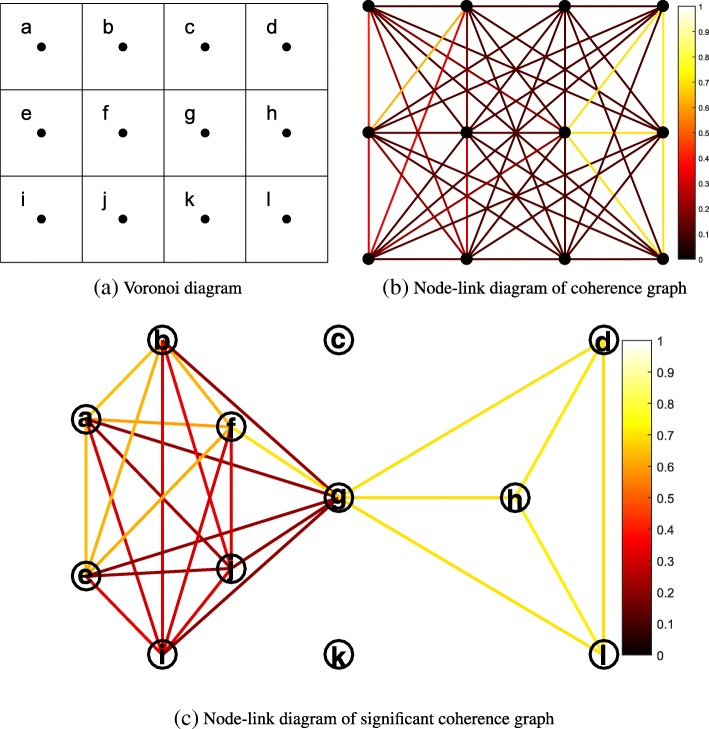
Example layout of a synthetic coherence network with coherence matrix as shown in Table 1. **a** Voronoi diagram showing vertex positions with vertex labels within the cells and Voronoi connections between vertices. Vertices are spatial neighbors if they are 4-connected, e.g., the spatial neighbors of vertex *f* are vertices, *b*, *e*, *g*, and *j*. **b** Layout preserving the vertex positions. Vertices are represented by solid circles and lines represent coherences between vertices, while the coherence values are encoded by the color of the lines (see the color bar on the right). **c** Manual layout of the significant coherence graph without considering the vertex positions

Functional brain connectivity obtained from EEG data is represented by an undirected *coherence graph* *G*=(*V*,*E*), defined by a set of vertices *V* and a set of edges *E*⊆*V*×*V* where vertices represent electrodes. Since weak coherences may represent spurious connections and these connections tend to obscure the topology of strong and significant connections (Rubinov and Sporns 2010), we only consider coherences with values above a pre-defined significance threshold (Halliday et al. 1995; ten Caat et al. 2008). Coherences above the significance threshold are represented by edges, whereas coherences below the threshold are ignored (see Fig. 3c). Vertices are not self-connected. To determine spatial relationships between electrodes, a Voronoi diagram is employed, which partitions the plane into regions of points with the same nearest vertex (for a simple example, see Fig. 3a. For EEG data, the vertex set is equal to the set of electrode positions. The vertices are referred to as (Voronoi) centers, and the region boundaries as (Voronoi) polygons. The area enclosed by a polygon is called a (Voronoi) cell. We call two cells *Voronoi neighbors* if they have a boundary in common. A collection of cells is called Voronoi connected if for a pair *ϕ*_0_,*ϕ*_*n*_∈*C*, there is a sequence *ϕ*_0_,*ϕ*_1_,...,*ϕ*_*n*_ of cells in *C*, with each pair *ϕ*_*i*−1_,*ϕ*_*i*_ consisting of Voronoi neighbors. Cells, vertices, nodes, and electrodes are interchangeable in this paper.

**Table 1:** Synthetic coherence matrix

	a	b	c	d	e	f	g	h	i	j	k	l
a	0	**0.65**	0.10	0.10	**0.64**	**0.60**	**0.20**	0.10	**0.30**	**0.23**	0.10	0.10
b	**0.65**	0	0.10	0.10	**0.63**	**0.63**	**0.21**	0.10	**0.32**	**0.33**	0.10	0.10
c	0.10	0.10	0.10	0	0.10	0.10	0.10	0.10	0.10	0.10	0.10	0.10
d	0.10	0.10	0.10	0	0.10	0.10	**0.70**	**0.71**	0.10	0.10	0.10	**0.70**
e	**0.64**	**0.63**	0.10	0.10	0	**0.62**	**0.20**	0.10	**0.33**	**0.20**	0.10	0.10
f	**0.60**	**0.63**	0.10	0.10	**0.62**	0	**0.70**	0.10	**0.30**	**0.31**	0.10	0.10
g	**0.20**	**0.21**	0.10	**0.70**	**0.20**	**0.70**	0	**0.69**	**0.20**	**0.20**	0.10	**0.70**
h	0.10	0.10	0.10	**0.71**	0.10	0.10	**0.69**	0	0.10	0.10	0.10	**0.72**
i	**0.30**	**0.32**	0.10	0.10	**0.33**	**0.30**	**0.20**	0.10	0	**0.32**	0.10	0.10
j	**0.23**	**0.33**	0.10	0.10	**0.20**	**0.31**	**0.20**	0.10	**0.32**	0	0.10	0.10
k	0.10	0.10	0.10	0.10	0.10	0.10	0.10	0.10	0.10	0.10	0	0.10
l	0.10	0.10	0.10	**0.70**	0.10	0.10	**0.70**	**0.72**	0.10	0.10	0.10	0

The rows and columns represent the nodes, and the cells contain the coherence values between nodes. Values above or equal to the significance threshold, in this case 0.2, are indicated in bold

### Community clique detection

We now introduce the CCB approach to find dense spatially-connected cliques from an EEG coherence network based on community structure. Such a clique is a set of electrodes that are spatially connected, and signals recorded by these electrodes are more densely connected within the same clique than with signals that are recorded by electrodes in other cliques.

#### Community structure

The community structure of a network is defined as groups of nodes with a high density of within-group connections and a lower density of between-group connections. Such structures have been observed in many different types of networks including social, biological, and tele-communication networks (Danon et al. 2005; Newman 2006). In particular, the community structure of a brain functional connectivity network shows the groups of neuronal areas where there is more synchronous activity within a group and less synchronous activity between groups. These communities may be considered as functional areas in the brain (Ahmadlou and Adeli 2011).

Various algorithms have been proposed for the identification of community structure from complex networks. Many of these algorithms are based on the idea of optimizing the so-called modularity index *Q* of the partition of a network (Newman and Girvan 2004; Newman 2006). In the case of a weighted network, this index is defined as follows (Blondel et al. 2008):

1 $$ Q= \frac{1}{2m}\sum_{v, v^{\prime}} \left[c(v, v^{\prime}) - \frac{K_{v}K_{v^{\prime}}}{2m}\right]\delta (L(v), L(v^{\prime})) {,}  $$

where *c*(*v*,*v*^′^) represents the weight (in our case the coherence value) of the edge between nodes *v* and *v*^′^, $K_{v} = \sum _{l}c(v,l)$ is the sum of weights of the edges incident to vertex *v*, *L*(*v*) is the community label of vertex *v*, the *δ*−function *δ*(*i*,*j*) is 1 if *i*=*j* and 0 otherwise, and $m=\frac {1}{2}\sum _{v, v'}c(v, v')$. The modularity *Q* is equal to the fraction of the sum of weights of edges that connect nodes in the same community minus what that fraction would be on average if the communities remained fixed but the edge weights were randomly distributed. The higher the value of *Q*, the more confident one can be that a significant community structure has been found (Newman 2006). So, the procedure of detecting community structure is usually based on maximizing the modularity index *Q*.

The optimal community structure for a given network is typically estimated with optimization algorithms rather than computed exactly (Danon et al. 2005; Rubinov and Sporns 2010)). A simple and efficient method of optimizing modularity was proposed by Blondel et al. (2008). It involves the local movement of nodes, and proceeds in two phases. In the first phase, each node of the network is initialized as a singleton community. Then, for each node *v*, the modularity *gain* *ΔQ* is evaluated that would result from removing *v* from its community *C*_*L*(*v*)_ and placing it in one of the other communities. The node *v* is then placed in the community for which this gain is maximum and positive. If no positive gain is possible, nothing is done. This process is applied repeatedly and sequentially for all nodes until no further improvement can be achieved and the first phase is then complete.

Equation 2 can be used to calculate the modularity gain *ΔQ* when removing one node *v* from its community *C*_*L*(*v*)_ to an arbitrary community *C*_*i*_ (Blondel et al. 2008; Rubinov and Sporns 2010; Sun et al. 2009):

2 $$ \Delta Q = \frac{1}{2m} \left(\sum_{l \in C_{i}} c(v, l) - \sum_{l \in C_{L(v)}} c(v, l) - \frac{K_{v}\left(K_{C_{i}} - K_{C_{L(v)}} + K_{v}\right)}{2m} \right){,}   $$

where $K_{C_{L(v)}}$ is the sum of the weights of the links incident to nodes in *C*_*L*(*v*)_, $K_{C_{i}}$ is the sum of the weights of the links incident to nodes in *C*_*i*_, and *K*_*v*_ is the sum of the weights of the links incident to node *v*.

The second phase of the algorithm consists of building a new network whose nodes are the communities found in the first phase. To do so, the weights of the links between the new nodes are given by the sum of the weights of the links between nodes in the corresponding two communities. Links between nodes of the same community lead to self-loops for this community in the new network. Once this second phase is completed, the first phase of the algorithm is reapplied to the new network. The combination of both phases is called a “pass”. The passes are iterated until there are no more changes.

#### Community clique detection method

Here, we extend the method proposed by Blondel et al. (2008) to obtain dense spatially-connected cliques, the community clique, consisting of Voronoi-connected vertices of the EEG coherence network.

The outline of our method can be summarized as follows. The difference with Blondel’s method is in step 2, the calculation of the modularity gain, where an extra condition is applied which ensures that the resulting communities are spatially connected cliques (see the introduction for the motivation):

1. Assign a unique community to each node of the network.
2. Use 2 to calculate the modularity gain *ΔQ* for node *v* caused by removing node *v* from its community and placing it in another community *such that the node v is connected to each node of that community and has at least one Voronoi neighbour in that community*.
3. Place the node *v* in the community for which the gain is the highest and positive. If no positive gain is available, nothing is done.
4. Continue repeating steps (2) and (3) until every node is processed.
5. Repeat steps (2) -(4) until no further improvement of the modularity index *Q* is achieved.

Note that the algorithm’s output depends on the order in which the nodes are processed in step (2). The ordering does not have a significant influence on the modularity that is obtained, but can influence the computation time (Blondel et al. 2008). In our case, a decreasing order is chosen based on the average local coherence of vertices, which is also used to detect basins in the IWB method (ten Caat et al. 2008).


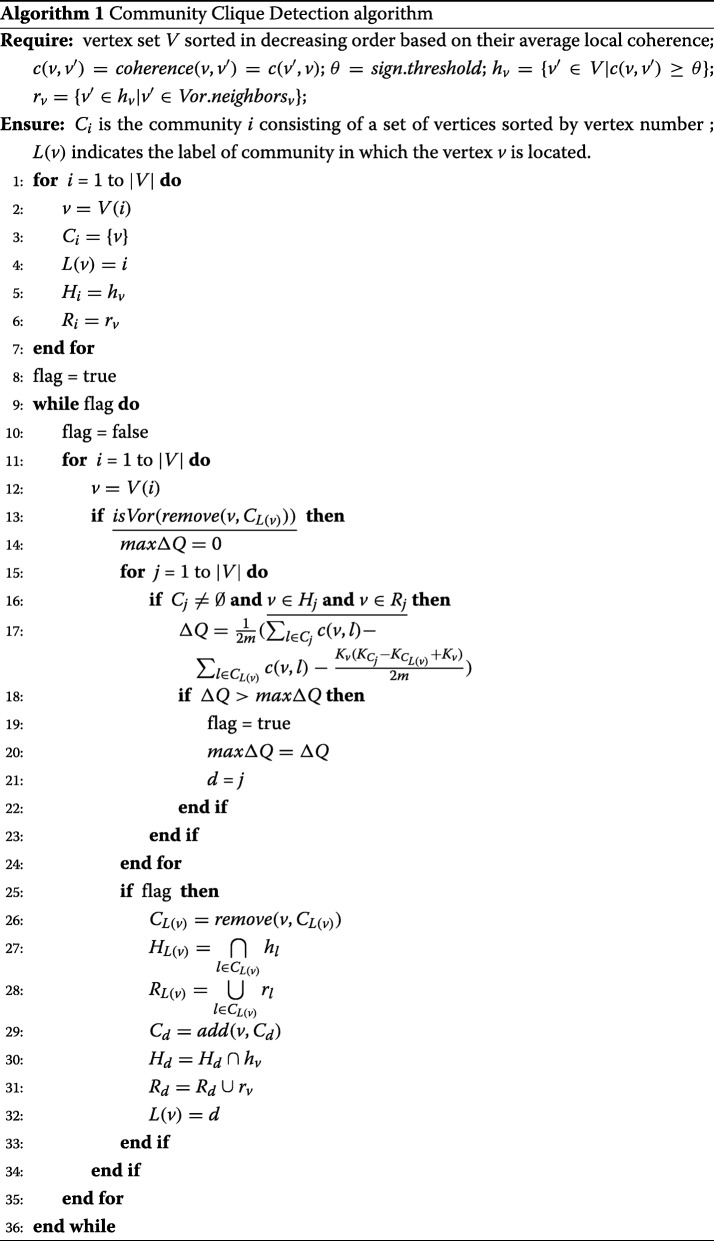


Algorithm 1 shows the pseudocode of the community clique detection procedure. It maintains the following dynamic vertex sets in the coherence graph of significant electrode connections:

- *C*_*i*_ contains a sorted list of the vertices in community *i*;
- *L*(*v*) is the community label of vertex *v*;
- *H*_*i*_ contains a list of vertices (sorted by vertex number) that are connected to *each* of the vertices in *C*_*i*_;
- *R*_*i*_ contains a list of vertices which have at least one Voronoi neighbor in *C*_*i*_.

The operation *remove*(*v*,*C*_*L*(*v*)_) removes the node *v* from the community *C*_*L*(*v*)_ and returns a set consisting of the remaining nodes. Similarly, *add*(*v*,*C*_*i*_) inserts node *v* into the community *C*_*i*_ and returns the updated community. The operation *isVor*(*C*_*i*_) returns “true” if the community *C*_*i*_ is empty, or if it only has one vertex, or if each pair of vertices in *C*_*i*_ is Voronoi-connected when |*C*_*i*_|>1; and it returns “false” if not. The size of a vertex set is denoted by |·|.

We now turn to a more precise analysis of the algorithm:

- *Initialization (lines 1-7).*
  - Initially, every node of the network is a singleton community (line 3).
  - The set of vertices which are connected to vertices of community *C*_*i*_ is identical to the set of vertices which are connected to the node *v*=*V*(*i*) (line 5)
  - The set of vertices which are connected Voronoi-neighbours of vertices of community *C*_*i*_ is the set of vertices that are connected Voronoi-neighbours of the node *v*=*V*(*i*) (line 6).
  - Set *flag* as *true* (line 8). *flag* is used to indicate that the modularity can be improved. If *flag* is *false*, there is no improvement of modularity.
- *Main Procedure (line 8-line 37).* This consists of the following steps.
  - Take the *i*th node *v*=*V*(*i*) from *V*. *If after removing v from its original community C*_*L*(*v*)_* any pair of remaining vertices is not Voronoi-connected, nothing is done, and the procedure continues with a new node (line 13).* Otherwise, set the maximal gain of modularity *maxΔQ* to zero, and take the *j*th community *C*_*j*_.
  - *In case C*_*j*_* is empty or v is not connected to any nodes of C*_*j*_* or v has no Voronoi neighbors in C*_*j*_*, nothing is done, and the procedure continues with a new community (line 16).* Otherwise, compute the modularity gain *ΔQ* (line 17).
  - If the current gain *ΔQ* is higher than *maxΔQ*, which means the modularity can be improved, set the label of the current community *j* to the destination community label *d* to which community the node *v* will move (line 21). Otherwise, nothing is done, and the procedure continues with a new community.
  - After all communities are traversed, select the first community which has the highest *ΔQ*, and update community *C*_*L*(*v*)_ by removing node *v* from its original community *C*_*L*(*v*)_ (line 26); replace *H*_*L*(*v*)_ by vertices that are connected to each node of the updated community *C*_*L*(*v*)_ (line 27); replace *R*_*L*(*v*)_ by the vertices that are connected Voronoi neighbours of nodes of the updated community *C*_*L*(*v*)_ (line 28); move node *v* into the destination community *C*_*d*_ (line 29); replace *H*_*L*(*v*)_ by its intersection with the set of vertices that are connected with *v* in the coherence graph (line 30); replace *R*_*L*(*v*)_ by its union with the vertices that are connected Voronoi neighbours of *v* (line 31); *v* receives label *d* (line 32).
  - This procedure is repeated until no improvement is obtained, which means *flag*=*false* after all nodes have been processed.

**Fig. 4:**
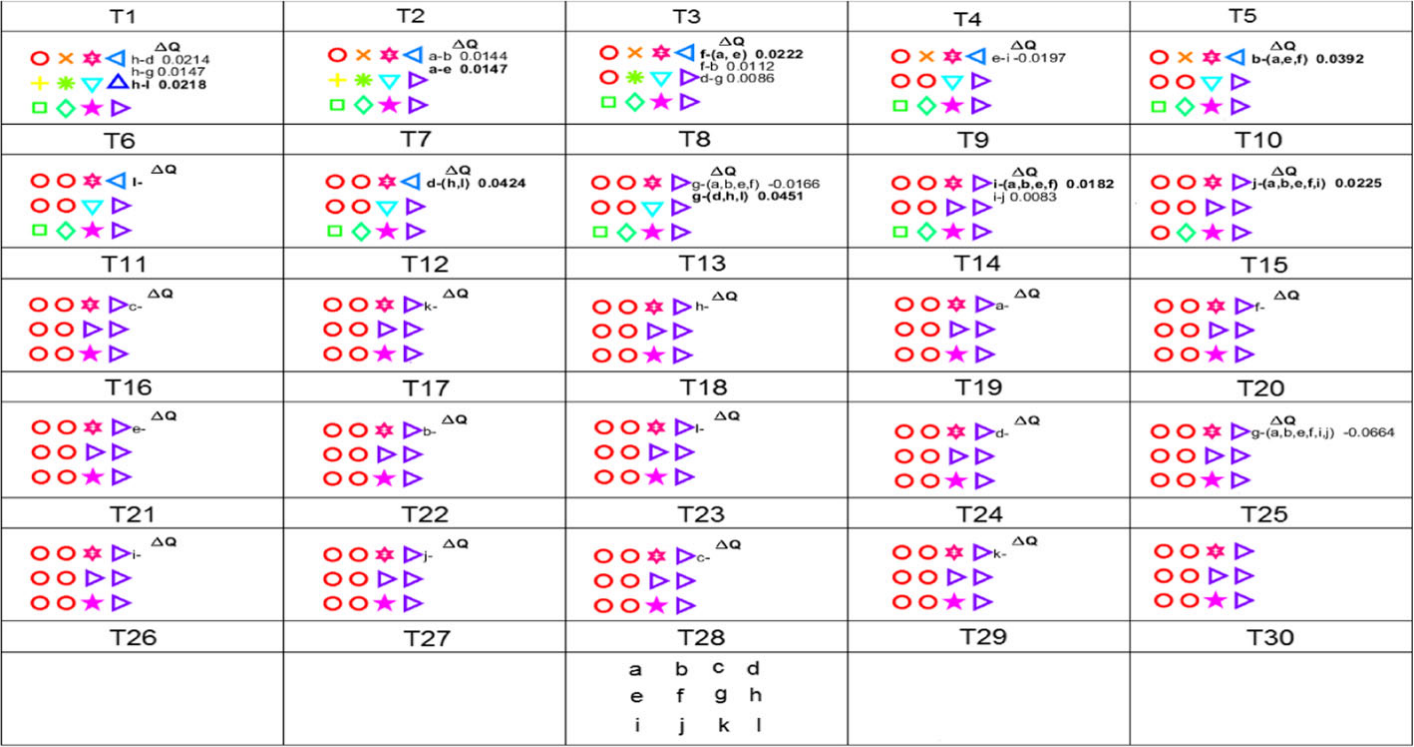
Illustration of Voronoi-connected community clique detection for a coherence network with the coherence matrix shown in Table 1. Each colored symbol represents a community at **T1**. For each step **Ti**, the gain *ΔQ* is shown on the right. Before the dash: the node to be removed; after the dash: the node or nodes constituting a community. **T28** shows the vertex positions in 2D space

Figure 4 illustrates the procedure of community clique detection for an EEG coherence network, with the coherence matrix shown in Table 1. Table 1 shows a synthetic coherence network, and we use it to illustrate the procedure of the three methods. The following detailed description contains references to Fig. 4. For a dataset of 119 electrodes, the computing time was around 0.84 s on a modern desktop computer (Intel 3.2 GHz, 8 GB RAM).

At **T1**, the initial stage, each of these twelve vertices correspond to a unique community represented by a specific colored symbol: *L*(*a*)=1, *L*(*b*)=2, *L*(*c*)=3, *L*(*d*)=4, *L*(*e*)=5, *L*(*f*)=6, *L*(*g*)=7, *L*(*h*)=8, *L*(*i*)=9, *L*(*j*)=10, *L*(*k*)=11, *L*(*l*)=12. Then, we calculate the modularity gain *ΔQ* caused by removing *k* (since *k* has the highest local average coherence, and the descending order of vertices based on their local average coherence is: *h*,*a*,*f*,*e*,*b*,*l*,*d*,*g*,*i*,*j*,*c*,*k*) from its community to the other communities; all the values of *ΔQ* are listed on the right in Fig. 4.

**T2-T12.** At every next step, the next vertex *v* will be chosen and the gain of removing *v* from its community *C*_*L*(*v*)_ to the remaining communities will be computed. If the positive highest gain *maxΔQ* results from the movement of node *v* to the community in which *v* has at least one Voronoi neighbour and is connected with each vertex in that community, then the vertex *v* will be removed from its original community to the destination community *C*_*Des*_, *C*_*L*(*v*)_ is updated by deleting *v* from *C*_*L*(*v*)_ and *C*_*Des*_ is replaced by the union of itself with *v*. At **T2**, *v*=*h*, *C*_*L*(*v*)_=*C*_8_=*∅*, *C*_*Des*_=*C*_12_={*h*,*l*}. At **T3**, *v*=*a*, *C*_*L*(*v*)_=*C*_1_=*∅*, *C*_*Des*_=*C*_5_={*a*,*e*}. At **T4**, *v*=*f*, *C*_*L*(*v*)_=*C*_6_=*∅*, *C*_*Des*_=*C*_5_={*a*,*e*,*f*}. At **T5**, *v*=*e*, nothing is done since *maxΔQ* is negative when merging *e* and *i* into one community. At **T6**, *v*=*b*, *C*_*L*(*v*)_=*C*_2_=*∅*, *C*_*Des*_=*C*_5_={*a*,*b*,*e*,*f*}. At **T7**, *v*=*l*, nothing is done since all the communities except *C*_12_, the original community, have no connected Voronoi neighbours of *l*. At **T8**, *v*=*d*, *C*_*L*(*v*)_=*C*_4_=*∅*, *C*_*Des*_=*C*_12_={*d*,*h*,*l*}. At **T9**, *v*=*g*, *C*_*L*(*v*)_=*C*_7_=*∅*, *C*_*Des*_=*C*_12_={*g*,*d*,*h*,*l*}. At **T10**, *v*=*i*, *C*_*L*(*v*)_=*C*_9_=*∅*, *C*_*Des*_=*C*_5_={*a*,*b*,*e*,*f*,*i*}. At **T11**, *v*=*j*, *C*_*L*(*v*)_=*C*_10_=*∅*, *C*_*Des*_=*C*_5_={*a*,*b*,*e*,*f*,*i*,*j*}. At **T12**, *v*=*c*, and at **T13**, *v*=*k*, nothing is done since the vertex *v* has no connected Voronoi-neighbours.

From **T14** on, all vertices will be traversed again. The gain *ΔQ* can be easily computed and it can be observed that there is no more positive gain, which means the modularity can not be improved anymore. So the detection procedure stops. Finally, we obtain two community cliques {*a*,*b*,*e*,*f*,*i*,*j*} and {*g*,*d*,*h*,*l*} at **T25**.

### FU detection using the MCB and IWB method

#### MCB method

The maximal clique based (MCB) method (ten Caat et al. 2007a) is an extension of the method proposed by Bron and Kerbosch (1973). It detects maximal cliques consisting of Voronoi-connected vertices. Its recursive procedure maintains four dynamic vertex sets (ten Caat et al. 2008):

- The set *compsub* contains an increasing or decreasing clique.
- The set *currentcand* contains the candidates that are a Voronoi neighbor of at least one element in *compsub*, and only these can be added to *compsub* at the current step.
- The set *complcand* is the complement of *currentcand* in *candidates* containing vertices that are connected to all vertices in *compsub*.
- The set *not* contains vertices that are connected to all vertices in *compsub* and that were added to *compsub* previously.

At each call, the element from *currentcand* that has the largest number of connections with the other candidates (*currentcand*∪*complcand*) is added to *compsub*. Let this element be *v* (in the coherence graph). The set *newcurrentcand* is the intersection of *currentcand* and the neighborhood of *v* (in the coherence graph), united with the Voronoi neighbors of *v* in *complcand*. The set *newcomplcand* is the intersection of *complcand* and the neighborhood of *v* (in the coherence graph), minus the Voronoi neighbors of *v* in *complcand*. The set (*new*)*not* is the intersection of *not* and the neighborhood of *v*. This is repeated until *newcurrentcand* is empty. If *newnot* is also empty, then *compsub* is a Voronoi-connected maximal clique.

**Fig. 5:**
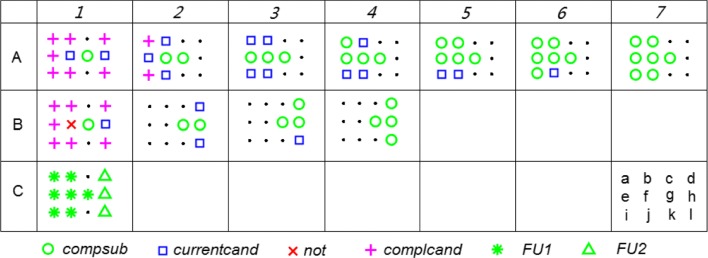
Illustration of Voronoi-connected maximal clique detection using the MCB method for a coherence network with the coherence matrix shown in Table 1 and associated adjacency matrix shown in Table 2. **C7** illustrates the vertex positions in the 2D space. Compare to Fig. 4

Figure 5 illustrates Voronoi-connected maximal clique detection using the MCB method for a coherence network, with the coherence matrix shown in Table 1. Its adjacency matrix is shown in Table 2 (here, we set the threshold at 0.2), and the Voronoi diagram is shown in Fig. 3a.

**Table 2:** Adjacency matrix

	a	b	c	d	e	f	g	h	i	j	k	l
a	0	1	0	0	1	1	1	0	1	1	0	0
b	1	0	0	0	1	1	1	0	1	1	0	0
c	0	0	0	0	0	0	0	0	0	0	0	0
d	0	0	0	0	0	0	1	1	0	0	0	1
e	1	1	0	0	0	1	1	0	1	1	0	0
f	1	1	0	0	1	0	1	0	1	1	0	0
g	1	1	0	1	1	1	0	1	1	1	0	1
h	0	0	0	1	0	0	1	0	0	0	0	1
i	1	1	0	0	1	1	1	0	0	1	0	0
j	1	1	0	0	1	1	1	0	1	0	0	0
k	0	0	0	0	0	0	0	0	0	0	0	0
l	0	0	0	1	0	0	1	1	0	0	0	0

The matrix element is 1 if the corresponding coherence value (Table 1) is above or equal to the threshold (0.2), otherwise the element is set to 0

The following detailed description contains (row and column) references to Fig. 5. The procedure **A.** starts with all twelve vertices in the set candidates (not illustrated), and with *not*=*∅*. **A1.** Then, the vertex *g* with the highest degree (following Table 2) is first added to *compsub*. Its adjacent vertices in the coherence graph are in *currentcand* if they are spatial neighbours ({*f*,*h*}); otherwise, they are in *complcand* ({*a*,*b*,*d*,*e*,*i*,*j*,*l*}). **A2-A6.** At every step, the element, say, *v*, from *currentcand* that has the largest number of connections in the coherence graph with the other candidates (*currentcand*∪*complcand*) is added to *compsub*. In case of ties, one vertex is selected randomly. The spatial neighbours of *v* in *complcand*, denoted by *Λ*(*v*), are moved from *complcand* to *currentcand*. Furthermore, vertices not adjacent to *v* in the coherence graph, denoted by *Γ*^*c*^(*v*), are removed from both *currentcand* and *complcand*. This continues until *currentcand* is empty. At **A2**, *v*=*f*, *Λ*(*v*)={*b*,*e*,*j*}, and *Γ*^*c*^(*v*)={*d*,*h*,*l*}. At **A3**, *v*=*e*, *Λ*(*v*)={*a*,*i*}, and *Γ*^*c*^(*v*)=*∅*. At **A4**, *v*=*a*, *Λ*(*v*)=*∅*, and *Γ*^*c*^(*v*)=*∅*. At **A5**, *v*=*b*, *Λ*(*v*)=*∅*, and *Γ*^*c*^(*v*)=*∅*. At **A6**, *v*=*i*, *Λ*(*v*)=*∅*, and *Γ*^*c*^(*v*)=*∅*. At **A7**, *v*=*j*, *Λ*(*v*)=*∅*, *Γ*^*c*^(*v*)=*∅*, and *compsub*={*a*,*b*,*e*,*f*,*i*,*j*,*g*} is a Voronoi-connected maximal clique, because *currentcand*=*∅* (and *not*=*∅*).

**B.** A later iteration for the MCB method returns to the situation preceding **A2**, with vertex *g* in the *compsub*, and vertex *f* put into the set of *not*. Then, at **B1.** only the vertex *h* is retained in *currentcand*, and *complcand* is the same as in **A1.** ({*a*,*b*,*e*,*i*,*j*,*d*,*l*}). At **B2**, *v*=*h*, *Λ*(*v*)={*d*,*l*}, *Γ*^*c*^(*v*)={*a*,*b*,*e*,*i*,*j*}, and *not*=*∅*. At **B3**, *v*=*d*, *Λ*(*v*)=*∅*, *Γ*^*c*^(*v*)=*∅*, and *not*=*∅*. At **B4**, *v*=*l*, *Λ*(*v*)=*∅*, *Γ*^*c*^(*v*)=*∅*, *not*=*∅*, and *compsub*={*g*,*d*,*h*,*l*} is a Voronoi-connected maximal clique, because *currentcand*=*∅* (and *not*=*∅*).

As it can be seen from the above example, every vertex can be part of multiple (Voronoi-connected) maximal cliques. (In the above example, vertex *g* belongs to Voronoi-connected maximal cliques {*a*,*b*,*e*,*f*,*i*,*j*,*g*} and {*g*,*d*,*h*,*l*}). To assign a unique label to every vertex, a quantity *total strength* is defined for a (sub)graph *G*=(*V*,*E*) as the sum of all edge values. For a vertex detected in more than one clique, it will be assigned to the clique which has the largest *total strength*. Then, the final cliques in above example are *{a, b, e, f, i, j, g}*and *{d, h, l }* (see **C1**).

#### IWB method

The IWB method is an alternative to the MCB method. It is a greedy method, approximating Voronoi connected maximal cliques on the basis of an edge-based watershed transform (ten Caat et al. 2007b).

This IWB method contains two main steps:

- *Initialization*. An edge *queue* is initialized with edges (corresponding with a significant coherence) between markers, which are defined as nodes having locally maximal average coherence, and their Voronoi neighbors. These edges are sorted in a descending order based on their values. Each marker corresponds to a basin and is assigned a unique label.
- *Main Procedure*. Remove the first edge, *e*=(*v*,*v*^′^), from the *queue*, and determine the label of node *v*^′^. In case the node *v*^′^ is unlabelled, *v*^′^ receives the label of *v* and the *queue* is extended with the edges between *v*^′^ and its unlabelled connected Voronoi-neighbours, if *v*^′^ is connected to every node of the basin where *v* is in. In case *v*^′^ was also labelled, check if the two basins that contain *v* and *v*^′^ can merge into a single basin. If so, then merge them, otherwise nothing is done.

The main procedure is repeated until *queue* is empty. Each basin then corresponds to an FU^IWB^.

**Fig. 6:**
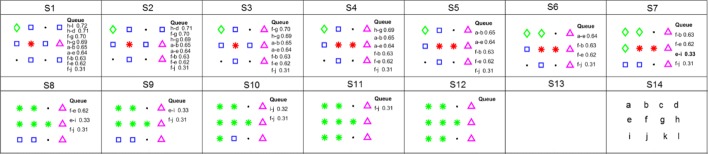
Illustration of FU detection using the IWB method for a coherence network with the coherence matrix shown in Table 1. Three basins are represented by a green diamond, red star, and magenta up-triangle, respectively. Blue squares represent vertices that have at least one significant edge in the *queue* connected to a labelled vertex. The edges in *queue* and their values are listed (in descending order) on the right for each step. The new inserted edges are indicated by bold text, e.g., “e-i” which appears at **S7**. **S14** illustrates the vertex positions in the 2D space

Figure 6 illustrates FU detection with the IWB method for a coherence network, with the coherence matrix shown in Table 1 and the Voronoi diagram shown in Fig. 3a. The following detailed description contains references to Fig. 6.

**Fig. 7:**
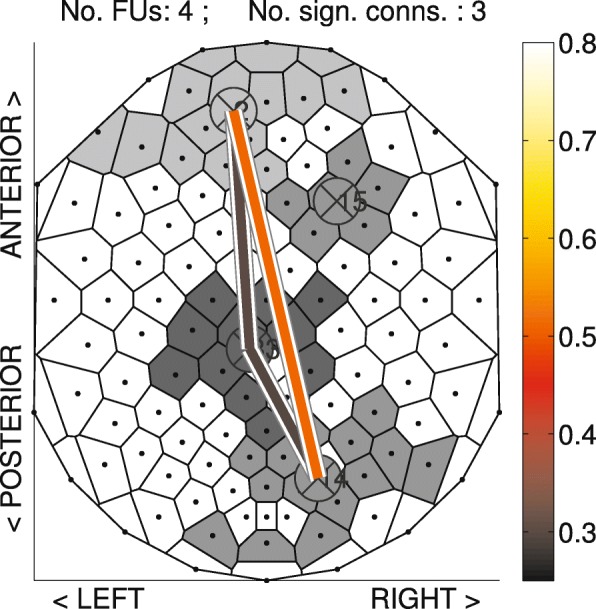
Example of an FU^MCB^ map (ten Caat 2008) as obtained during an auditory oddball task (see “Experimental setup” section. Spatial groups of similarly colored (in gray scale) cells correspond to FUs with a size of at least four, while white cells are part of smaller FUs. Circles overlayed on the cells represent the barycenters of the FUs and are connected by lines whose color reflects the average coherence between all electrodes of the FUs (see color bar). See ten Caat et al. (2007a) for details

At **S1**, three markers, *a*,*f*,*h*, are detected, and they are represented by a green diamond, red star, and magenta up-triangle, respectively. Each is then assigned a unique number: *L*(*a*)=1, *L*(*f*)=2, *L*(*h*)=3; edges (corresponding to significant coherences) between markers and their unlabelled Voronoi neighbours represented by blue squares are added in the *queue*; the edge with the highest value in the *queue* is *c*(*h*,*l*) and is shown at the top on the right of **S1**.

**S2-S11.** At every next step, the edge, say, *c*(*v*,*v*^′^), with the highest value in the queue is removed. Then, the vertex *v*^′^ is labelled. The edges (denoted by *Φ*(*v*^′^)) between *v*^′^ and its unlabelled Voronoi-neighbours are inserted in the *queue* and will be highlighted in bold. At **S2**, *v*=*h*, *v*^′^=*l*, *L*(*v*^′^)=*L*(*h*)=3, and *Φ*(*v*^′^)=*∅*. At **S3**, *v*=*h*, *v*^′^=*d*, *L*(*v*^′^)=*L*(*h*)=3, and *Φ*(*v*^′^)=*∅*. At **S4**, *v*=*f*, *v*^′^=*g*, *L*(*v*^′^)=*L*(*f*)=2, and *Φ*(*v*^′^)=*∅*. At **S5**, *v*=*h*, *v*^′^=*g*, *h* which was labelled already in the previous stage. But, the basin of *g* and the basin of *h* can not merge since their union is not a clique in the coherence graph (*h*,*d*,*l* are not significantly connected with *f*, which can be derived from 1). Hence, nothing will be done at this stage. At **S6**, *v*=*a*, *v*^′^=*b*, *L*(*v*^′^)=*L*(*a*)=1, and *Φ*(*v*^′^)=*∅*. At **S7**, *v*=*a*, *v*^′^=*e*, *L*(*v*^′^)=*L*(*a*)=1, and *Φ*(*v*^′^)={(*e*,*i*)}. At **S8**, *v*=*f*, *v*^′^=*b*, *b* was labelled already in the previous stage. But the basins of *f* and *b* can merge since their union is a clique in the coherence graph. Hence, vertices in the basin of *b* will be moved to the basin of *f*. *L*(*v*^′^)=*L*(*f*)=3, *L*(*a*)=*L*(*f*)=3, *L*(*e*)=*L*(*f*)=3, and *Φ*(*v*^′^)=*∅*. At **S9**, *v*=*f*, *v*^′^=*e*, *L*(*v*^′^)=*L*(*e*)=3, and *Φ*(*v*^′^)=*∅*. At **S10**, *v*=*e*, *v*^′^=*i*, *L*(*v*^′^)=*L*(*e*)=3, and *Φ*(*v*^′^)=*∅*. At **S11**, *v*=*i*, *v*^′^=*j*, *L*(*v*^′^)=*L*(*j*)=3, and *Φ*(*v*^′^)=*∅*. At **S12**, *v*=*f*, *v*^′^=*j*, *L*(*v*^′^)=*L*(*j*)=3, and *Φ*(*v*^′^)=*∅*. Now, two basins have been detected, *{ a, b, e, f, i, j, g}* and *{ d, h, l}*, because the *queue* is empty.

### FU visualization

Given FUs, the *inter-FU coherence*$c^{\prime }_{\lambda }$ at frequency *λ* between two FUs *C*_1_ and *C*_2_ is defined as the sum of coherence values between one vertex in *C*_1_ and the other vertex in *C*_2_, divided by the maximal number of edges between *C*_1_ and *C*_2_:

3 $$ c'_{\lambda}(C_{1}, C_{2}) = \frac{\sum_{i,j} \{ c_{\lambda} (v_{i}, v_{j})| v_{i} \in C_{1}, v_{j} \in C_{2} \} }{|C_{1}| |C_{2}|} {.}  $$

Note that coherences between *any* pair of vertices are taken into account to normalize for the size of the FUs even if their coherence is below the predefined threshold.

An FU map visualizes each FU as a set of Voronoi cells with identical gray values and with different gray values for adjacent FUs. See Fig. 7 for an example. The colour of the circle over the geographic centre of FU *C*_1_ reflects its *average coherence*$\hat {c}_{\lambda } (C_{1})$, which is defined as $\hat {c}_{\lambda } (C_{1}) = \frac {\sum _{i,j} \{ c_{\lambda } (v_{i}, v_{j})| v_{i} \in C_{1}, v_{j} \in C_{1} \} }{|C_{1}| (|C_{1}|-1)}$. Note that the geographic centre of an FU can be located in a cell not belonging to the corresponding FU. A line is drawn between FU centres if the corresponding inter-FU coherence exceeds a predefined threshold.

In our case, only FUs larger than five cells are considered. White Voronoi cells are part of smaller FUs.

### Comparison of methods applied to synthetic EEG coherence networks

In this subsection, we will first compare the proposed method with two other methods using a synthetic EEG coherence network (see Fig. 3 and Table 1). The comparison between the three methods applied to real functional brain networks is described in the “Results” section.

For the MCB method, at the stage of (Voronoi-connected) maximal clique detection, coherences are considered equally when their values are above or equal to the threshold. This method intends to find all possible maximal cliques in a coherence network. Then, at the stage of vertex assignment, the vertex is assigned to the clique which has the highest *total strength* when it is part of multiple cliques, no matter how strong the vertex is connected to these cliques. Hence, the vertex will be placed in the clique with more vertices when two cliques have an equal average coherence. For example, at the stage of maximal clique detection, we found two overlapping maximal cliques, {*a*,*b*,*e*,*f*,*i*,*j*,*g*} and {*g*,*d*,*h*,*l*}, in Fig. 5. But the former has a higher total strength, so *g* is removed from {*g*,*d*,*h*,*l*} at the assignment stage, and the final cliques are shown in **C1**.

For the IWB method, the edges connecting a labelled vertex and an unlabelled vertex will be placed in the queue first if these vertices are significantly connected Voronoi neighbours. Then, the unlabelled vertex will be placed in the clique in which one of its Voronoi neighbours has the highest coherence with this vertex compared to others in the queue. For example, at **S3** in Fig. 6, the vertex *g* is assigned to *f* since their connection value is 0.71 while the connection value with *h* is 0.69. The final cliques are shown in **S12**.

The community clique-based (CCB) method detects the dense spatially connected cliques in a coherence network. It first calculates the degree of connections between nodes and community cliques, which is quantified by modularity. Then, the node will be placed in the clique which has the strongest node-community connection. For example, at **T8** in Fig. 4, vertex *g* has a stronger connection with clique {*d*,*h*,*l*} than with clique {*a*,*b*,*e*,*f*}. At **T21**, vertex *g* still has a stronger connection with clique {*d*,*h*,*l*} than with clique {*a*,*b*,*e*,*f*,*i*,*j*}. The final cliques are shown in **T26**.

## Results

In this section, we will first describe the experimental setup, before applying the CCB method to twelve participants for an example application. To compare the three methods when applied to real EEG coherence networks, we selected four of these sixteen participants (two young and two old) to demonstrate any differences.

### Experimental setup

Brain responses were recorded during an auditory oddball detection experiment, in which older and younger participants were instructed to count target tones and ignore standard tones. After the experiment, each participant had to report the number of perceived target tones. In our data, brain responses to *L* target tones were analyzed in *L* segments of 1 second, sampled at 256Hz. A procedure from Neurospec was adopted to compute the coherence (www.neurospec.org). A detailed description of the procedure is given in ten Caat et al. (2008).

In the present study we do not consider ongoing EEG but the event-related potential (ERP) which is an EEG recording of the brain response to a sensory stimulus. To calculate the coherence for an ERP with *L* repetitive stimuli, the EEG data can be separated into *L* segments. A *significance threshold* for the estimated coherence is then given by Halliday et al. (1995):

4 $$ \theta = 1 - p^{1/(L-1)} {,}   $$

where *p* is a probability value associated with a confidence level *α*, such that *p*=1−*α*.

Throughout this section, we use *p*=0.01, and *L*=13 segments. In addition, we set the inter-FU coherence threshold to the same value as the significance threshold *θ*.

### Comparison of methods applied to real EEG coherence networks

**Fig. 8:**
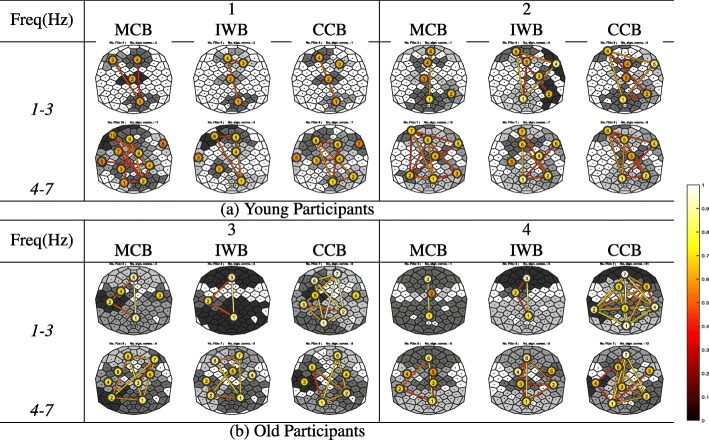
Illustration of FU maps (top view, nose on top) obtained by using the three FU detection methods for two different EEG frequency bands. **a** FU maps obtained from two young participants. **b** FU maps obtained from two older participants
- For the older participants, however, there are large differences between FU maps for the different methods. In the [4, 7]Hz frequency band, the three methods result in a similar number of FUs. In the [1, 3]Hz frequency band for participant *3* both MCB and IWB methods detect two large FUs located anteriorly and posteriorly: FUs^MCB^**1**, **5** and FUs^IWB^**1**, **3**. In the CCB method, however, these FUs are split into small FUs due to a weak inter-community connection. For example, FU^MCB^**5** splits into FUs^CCB^**6** and **7**, while FU^MCB^**1** splits into FUs^CCB^**1** and **2**. From these splits, we can see that FUs^CCB^**6** and **7** have higher average coherence than FU^MCB^**5**, and the inter-FU coherence between FUs^CCB^**6** and **7** is also lower than their average coherence. This is also true for FU^MCB^**1** and FUs^CCB^**1**, **2**. From a global point of view, FU^CCB^**7** has the highest average coherence, followed by **1** and **2**, and there are higher inter-FU coherences among these FUs. For participant *4*, the MCB method detects two large FUs located anteriorly and posteriorly, with significant inter-FU coherence between them. The IWB method has a similar result, except for the frontocentral connection. The CCB method, in contrast, finds a total of seven FUs with size above five. Compared to the CCB method, FU **1** obtained by the MCB and IWB methods is split into four FUs **1**, **2**, **3**, **4** in the CCB method due to the weak inter-community connections with each other. FU^CCB^**1** in the CCB method has the highest average coherence among these four FUs. From a global point of view, the two FUs^CCB^ having the strongest connection are **1** and **7**, which are located at the frontal and parietal-occipital areas of the brain, respectively. In the [4, 7]Hz frequency band, the MCB and IWB methods produce similar results, except in the frontocentral area of the brain. The main difference between methods is that FU^MCB^**1** splits into FUs^CCB^**1** and **2** in the CCB method due to weak inter-community connections. In addition, the average coherence of FU^CCB^**1** and **2** is higher than FU^MCB^**1**. FU^MCB^**6** is an extension of FU^CCB^**7**, but it can be seen that their average coherence differs.

Besides comparing the three methods when applied to the synthetic EEG network in the “Method” section, we also compared the three methods when applied to real EEG coherence networks as obtained from two younger and two older participants in the experiment described in “Experimental setup” subsection. The results are shown in Fig. 8, and we make the following observations.

- For the young participants, it can be observed that there is no big difference between FU maps obtained from these three methods. In the [1, 3]Hz frequency band, FU maps are very similar for both young participants in terms of, for example, the number of FUs and their location. Similarly, for the [4, 7]Hz frequency band, there are no big differences between the methods either.

In this example, the FU maps obtained from older participants generally have larger FUs and the coherence within-and between FUs is also high compared to the young participants. This could be interpreted as the older participants having higher local and global synchronization. All the methods are trying to find cliques and for the young participants brain areas are apparently less synchronized, which results in lower coherence. In this case we end up with small(er) FUs. This also explains why the FU maps obtained from the three methods are similar for the younger participants. For the older participants, there is apparently high synchronization between brain areas. In this case, more electrodes make up larger cliques. The MCB and IWB methods then detect larger FUs. Consequently, the detected FUs are less suited for analyzing local synchronization. In this case, however, the CCB method still considers the community properties: if an electrode can be put into several FUs, it will be added to the FU with which it has a strong connection rather than to the largest FU as in the other methods.

### FU^CCB^ maps

In this subsection, we apply (only) the CCB method to the data of six younger and six older participants in the experiment described in the “Experimental setup” subsection.

**Fig. 9:**
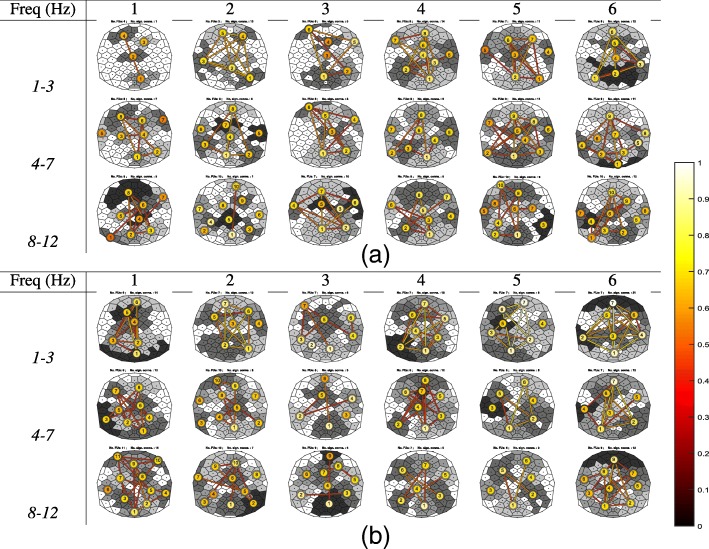
FU^CCB^ maps (|*FU*|≥5) for 6 younger adults and 6 older adults for three frequency bands (1-3, 4-7, and 8-12 Hz). Each FU is visualized as a set of Voronoi cells with identical gray values and with different gray values for adjacent FUs. White Voronoi cells are part of FUs, with |*FU*|<5. A line connects FUs if the inter-FU coherence exceeds the significance threshold, with its color depending on the value (see color bar, bottom right). The color of the circle located at the barycenter of each FU reflects the average coherence within FUs. **a** Young participants. **b** Old participants

Figure 9a and b show FU^CCB^ maps of the younger and older adults, respectively. In general, the color of circles and edges is lighter for older participants than for younger participants over the three frequency bands. This probably corresponds to earlier findings using a hypothesis-driven method (Maurits et al. 2006), indicating the older adults have higher local and global synchronization compared to the younger adults and that aging is associated with increased EEG coherence during a relatively easy cognitive task. The midline regions are usually less synchronized as reflected in the FUs of these regions being small and the color of circles and edges connecting with other FUs having darker colors. Throughout the three frequency bands, FUs with high average coherence (light circle color) are usually found in anterior and posterior regions, and these FUs usually have high inter-FU coherence which is also in accordance with previous observations in the literature (ten Caat et al. 2008), particularly for the older participants.

For the younger participants, the inter-FU coherence decreased with increasing frequency except for participant *1* (Fig. 9a). The left and right-temporal part of the brain are usually less synchronized compared to the older participants, for example, participants *1* and *3* have no left-temporal FUs while these FUs in the rest of young participants have a low average within-FU coherence. Participant *1* seems to be an exception in more ways, having the least synchronization among all FU maps for frequencies between 1-3 Hz and having only 4 FUs with a size above 5 and no lateral FUs for this frequency band.

For the older adults, the inter-FU coherence also decreased with increasing frequency, especially for the FUs located at the anterior and posterior regions, which can be derived from the color of the edge connecting these FUs fading (Fig. 9b). For example, it is very obvious for old participant *5*: the inter-FU coherence between FU^CCB^**7** and **1** for frequencies between 1-3 Hz is very high; the inter-FU coherence between FU^CCB^**6** and **1** for frequencies between 4-7 Hz is low, and the inter-FU coherence between FU^CCB^**6** and **1** for frequencies between 8-12 Hz is the lowest. There is also a variety in these findings among old adults, for example, the size of FUs for old participant *1* is usually smaller compared to other old participants and the color of the circle or edges is darker compared to the rest, as well. Another example is old participant *2* for frequencies between 4-7 Hz for which FUs **3**, **6** and **7** left- and right-temporally are small and not connected with other FUs, which means these regions are less synchronized and also are less synchronized with other regions.

## Conclusions and future work

Visualization is an important aspect in the analysis of EEG coherence, especially for multichannel EEG coherence networks. Since conventional methods either suffer from reduced spatial information or visual clutter, they have inherent limitations when applied to EEG coherence networks. We developed a visualization approach based on the functional unit (FU) concept that attempts to preserve spatial relationships between functional brain regions and allows analysis of functional connectivity within and between regions.

A new community clique based (CCB) method was proposed that first partitions an EEG coherence network into dense groups of spatially connected electrodes recording pairwise significantly coherent signals. The resulting communities (groups of electrodes) were visualized in an FU map, which makes it possible to investigate the relationship between functional brain connectivity and underlying brain structure. The novelty of this method is that it is helpful to analyze the local and global connectivity without any a priori hypotheses. Community cliques found by the CCB method can be used for further analysis, e.g., the analysis of ERP components across FUs and synchronization between FUs.

As topics for future work we first mention the influence of the order in which nodes are traversed in the CCB procedure, which needs to be further analyzed. Second, differences between FU maps were assessed only visually in our study. However, there is still the need to develop methods for comparing FU maps quantitatively, and to discriminate not only between single subjects, but also between different *groups*, e.g., old and young participants. Our method is a visually aided pre-processing method that can be used before analysis questions about data are well defined. Although our method is specific to EEG coherence networks, we believe that it can be easily adapted to other network visualizations which need to capture the whole structure of networks and that do not only depend on the analysis of single nodes or specified connections between pairs of nodes.

## Abbreviations

CCB: Community clique based
EEG: Electroencephalography
ERP: Event-related potential
FU: Functional unit
(I)WB: (Improved) watershed based
MCB: Maximal clique based
ROI: Regions Of interest
